# TipMT: Identification of PCR-based taxon-specific markers

**DOI:** 10.1186/s12859-017-1485-3

**Published:** 2017-02-11

**Authors:** Gabriela F. Rodrigues-Luiz, Mariana S. Cardoso, Hugo O. Valdivia, Edward V. Ayala, Célia M. F. Gontijo, Thiago de S. Rodrigues, Ricardo T. Fujiwara, Robson S. Lopes, Daniella C. Bartholomeu

**Affiliations:** 1 Laboratório de Imunologia e Genômica de Parasitos, Instituto de Ciências Biológicas, Universidade Federal de Minas Gerais, Belo Horizonte, Brazil; 2 Centro de Pesquisa René Rachou, Fiocruz, Belo Horizonte, Brazil; 3 Departamento de Computação, Centro Federal de Educação Tecnológica de Minas Gerais, Belo Horizonte, Minas Gerais Brazil; 4 Departamento de Computação, Universidade Federal do Mato Grosso, Barra do Garças, Mato Grosso Brazil

**Keywords:** Molecular marker, Specific primers, PCR, PCR Multiplex, Web application

## Abstract

**Background:**

Molecular genetic markers are one of the most informative and widely used genome features in clinical and environmental diagnostic studies. A polymerase chain reaction (PCR)-based molecular marker is very attractive because it is suitable to high throughput automation and confers high specificity. However, the design of taxon-specific primers may be difficult and time consuming due to the need to identify appropriate genomic regions for annealing primers and to evaluate primer specificity.

**Results:**

Here, we report the development of a Tool for Identification of Primers for Multiple Taxa (TipMT), which is a web application to search and design primers for genotyping based on genomic data. The tool identifies and targets single sequence repeats (SSR) or orthologous/taxa-specific genes for genotyping using Multiplex PCR. This pipeline was applied to the genomes of four species of *Leishmania* (*L. amazonensis, L. braziliensis, L. infantum* and *L. major*) and validated by PCR using artificial genomic DNA mixtures of the *Leishmania* species as templates. This experimental validation demonstrates the reliability of TipMT because amplification profiles showed discrimination of genomic DNA samples from *Leishmania* species.

**Conclusions:**

The TipMT web tool allows for large-scale identification and design of taxon-specific primers and is freely available to the scientific community at http://200.131.37.155/tipMT/.

**Electronic supplementary material:**

The online version of this article (doi:10.1186/s12859-017-1485-3) contains supplementary material, which is available to authorized users.

## Background

Polymerase chain reaction (PCR)-based typing methods are molecular diagnostic techniques widely used in biological and biomedical studies. The level of discriminatory power of PCR-based typing depends upon the molecular marker targeted. Therefore, identifying appropriate DNA target regions for primer annealing is a crucial step because these regions must be conserved within the target taxa but must vary among related taxa [1, 2].

Recent advances in next-generation sequencing technology are enabling genome sequencing projects at a significantly lower cost, even for non-model organisms. The resulting increase in the amount of genomic data available, combined with bioinformatics tools, have led to the identification of highly informative markers, such as microsatellites and orthologous or taxa-specific genes [3].

Microsatellites or single sequence repeats (SSR) are tandem repeated stretches of short nucleotide motifs, usually ranging from 1 to 6 bp, ubiquitously distributed in the genomes of eukaryotic organisms. These regions are more prone to genetic variation and the differences in the length of individual SSR loci can be easily screened by PCR. In fact, this technique has been useful for several studies including strain typing and population genetics [4, 5]. The conventional method of SSR discovery is time consuming and costly. Therefore, *in silico* mining analysis has been used to improve marker identification [6, 7].

Orthologs are homologous proteins in different species that evolved from a single ancestral sequence and are related by speciation events. These sequences tend to show more functional similarity than other homologs. The identification of orthologous genes is useful in a wide range of contexts, such as inference of gene function, comparative genomics, evolutionary conservation and sequence variability [8]. Due to their importance, many tools have been developed to predict ortholog groups, including the widely used software OrthoMCL [9].

The demand is increasing for bioinformatic tools that automate analysis of genomic data generated by next-generation sequencing technology [10]. An example is the development of automated procedures to facilitate species-specific primer design for diagnostic methods [2]. Several web-based tools for facilitating primer design are available [11], but many of them are written mainly to assist in the primer design process and are not meant to search for targets and analyze primer specificity. The use of fully automated methods to search for molecular markers and the availability of genomic data for a growing number of taxa would increase the efficiency of PCR-based genotyping applications. Moreover, this strategy might save time and resources because the *in silico* evaluation of the candidate primers against the target genomic sequence are performed prior to testing them in the laboratory [12]. Thus, there is a need for a tool to search for appropriate genomic target regions and then design specific primers towards the selected markers.

In this context, we have developed TipMT to meet the growing demand for easy-to-use software that facilitates the design of primers that target molecular markers to distinguish the genomic sequences of different taxa. This program only requires genomic sequences of a target species and offers, as an output, specific primers for a given taxa.

## Implementation

### Method Summary

The aim of the software pipeline is to provide a set of primer pairs flanking polymorphic sequences to identify taxa among related species using PCR, given their genomic sequences. By taking advantage of sequence data from related species, the pipeline identifies orthologous and singleton (taxa-specific) genes or SSR regions as target sequences that are likely to identify unique taxon or taxa. Because primer specificity is a key step in a PCR reaction, the pipeline identifies all potential annealing sites for the primers selected based on alignment and thermodynamics. Then, the TipMT evaluates compatibility among specific primers for designing multiplex PCR reactions. Finally, the program generates a virtual gel with the result of a simulated standard or multiplex PCR assay, where taxa can be identified by the size variation of the predicted PCR products.

**Fig. 1:**
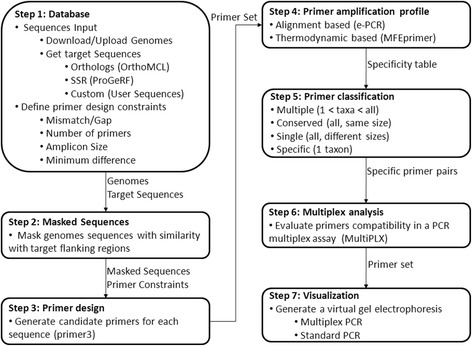
Flowchart for TipMT analysis

The program workflow is shown in Fig. 1 and consists of the following steps: 1) the database with data required for the next steps is generated. In this step, the user provides the genomic sequences and defines the type of target sequences, orthologous genes or microsatellites, which are then extracted from each taxon; in this step, the user also defines the primer design constraints; 2) regions of target sequences with similarity to the other genomic sequences provided by the user (cross reaction genomes) are masked; cross reaction genomes correspond to templates that should not cross-react in PCR assays; 3) candidate primers for each target sequence are designed; 4) the amplification profile of each taxon is obtained based on electronic PCR and the thermodynamic properties of the primers; 5) candidate primers are classified according to the number of taxa with amplicons; 6) the compatibility of specific primers in multiplex PCR reactions are checked; and 7) predicted PCR products for selected primers are visualized as virtual electrophoresis gels and analyzed.

### User Input Data

TipMT is flexible because it accepts three different input data as target sequences: genomes, predicted genes and user defined target sequences. The user may provide sequences by uploading files in FASTA format or entering Nucleotide Accession numbers, and then, corresponding sequences will be downloaded from the NCBI RefSeq database. There are two types of genomic sequences: target taxa and cross-reaction taxa. The former sequences will be used in the pipeline as templates for primer design and to check the specificity of the primers. The latter are sequences of species that should not cross-react during the PCR assays. Regions in the target taxa that have similarities to sequences in the cross-reaction taxa are not targeted during the primer-designing step. The number of taxa analyzed in TipMT is not limited, but the processing time is substantially increased for each taxon added. For example, in the test using *Leishmania braziliensis* and *Leishmania infantum* genomes as templates on “Ortholog target” mode run on a Intel(R) Xeon(R) X3430 2.40GHz, 4 CPUs and 8 GB RAM machine, the software spent 12 h, while the execution with *Leishmania amazonensis, L. braziliensis* and *L. infantum* genomes in the same mode run and machine spent 22 h.

The next step is the definition of the target sequences by the user, and each approach offers advantages. SSRs are highly polymorphic and tend to be conserved between closely related species [5]. These features enhance the success rate in the search for taxon specific primers. Moreover, SSR targets only require genomic sequences. Orthologs sequences are less error-prone than repetitive regions during genome assembly, thus the amplification failure tends to be lower compared to SSRs. Additionally, conserved primers among closely related taxa are easily found using this approach and singleton sequences enable the search for taxon-specific primers. In the case orthologs target sequences, the user should provide the sequences of the predicted genes in the genome. Also, users can have their custom sequences of interest be targets to design specific primers.

If the user chooses an SSR as a target, the pipeline will search SSR regions in the target genomes, and these regions will be the template sequences in the primer design step. On the other hand, if the user selects ‘ortholog target’, orthologous and singleton genes will be identified in the predicted coding regions provided by the user. Finally, the user may choose to provide target sequences instead of using the TipMT target search mechanism.

Lastly, the following primer design constraint parameters are defined: 1) mismatch and gap tolerance (both parameters are known to effect PCR specificity [12]); 2) PCR product sizes; 3) the number of primers per target (high values increase the chance to find specific primers but also increase the processing time); and 4) minimal difference (this value increases the PCR product size between taxa).

### Pipeline mechanism

TipMT is a web-based application that was written in Perl language. The client side was built primarily in HTML markup language with dynamic parts written in JavaScript programming language, and Java applets are used to input data and process files between the user and the application. The server side runs on PHP, and MySQL database is used to store the input parameters and results.

The pipeline uses Primer3 core [13] as the primer design engine and is built around other public domain programs, such as BEDtools [14], BioPerl [15], BLAST [16], EMBOSS [17], e-PCR [18], MFEprimer [19], MultiPLX [20], OrthoMCL [9] and ProGeRF [21]. These programs are used in the processing flow from raw sequences to the list of primers in the following list of procedures:

1. Target search. One way to improve sensitivity in PCR is to find the most appropriate template region for primer design. Ortholog and singleton sequences are identified in the predicted coding sequences provided by the user using OrthoMCL with default parameters values. ProGeRF searches for SSR regions, without degeneration or gaps in the sequence (perfect repeats).
2. Mask similar regions. Conservation of the flanking regions of the target sequences is essential for a high quality PCR assay because a high number of primer annealing sites can cause failure of the PCR assay [22]. Similar regions between target sequences and cross-reaction genomes are identified using a MEGABLAST search with default parameters. MEGABLAST was chosen due to its speed and its ability to handle slight differences in genomic sequences. Next, regions with more than 95% of identity are masked with lowercase nucleotides (initially, all sequences are set as uppercase in the database).
3. Primer design. Candidate primers are generated for each of the DNA template sequences using primer3 2.3.5 with default parameters values and an option that rejects the primer candidates with lowercase letters in the first 3’ end position. Because the high number of annealing sites influences primer specificity, this procedure decreases the rate of low-success specific primers, avoiding primer design in regions with similarity between species [23].
4. Specificity check. All candidate pairs of primers generated are evaluated for specificity using the alignment based e-PCR algorithm and by thermodynamic properties using MFEprimer software, with mismatch and gap tolerance chosen by the user. If both tools predict PCR products using the same pair of primers with same length, the pair of primers is selected for the next step.
5. Primer classification. The program recovers all potentially useful pairs of primers for the differentiation of taxa. If a pair of primers has only one amplification product in only one target genome, it is defined as ‘specific’. If a pair of primers has one amplification product in at least one other genome, it is defined as ‘multiple’. If it amplifies the same size products in all genomes, it is named as ‘conserved’, but if the PCR products have different sizes in all genomes, then it is a ‘single’ primer. ‘Single’ pairs of primers are capable of distinguishing all taxa in a simple PCR reaction because each has different sizes of amplification products in each genome.
6. Compatibility check. Specific primers are clustered into compatible groups for multiplexing PCR using MultiPLX, which tests all primer pairs for interactions, including dimer formation and differences in their melting temperatures.
7. Gel visualization. After a set of primers is chosen, the relative electrophoretic migration distances are calculated based on the expected length of the amplification products. Then, a virtual electrophoresis gel is generated showing the expected amplification profile as a result of a standard PCR assay using a mixture of target genomes as the template. Another virtual gel is generated for the amplification profile of the multiplex PCR reaction, to check for interactions among primers that generate undesired alternative products.

### *Pipeline* validation

This pipeline was validated using genomic sequences and genomic DNA samples from different species of the parasite *Leishmania*. Promastigote forms of *L. braziliensis* (MHOM/BR/75/M2904), *L. infantum* (MHOM/BR/74/PP75), *L. amazonensis* (IFLA/BR/67/PH8) and *L. major* (strain Friedlin) were cultured in Schneider’s insect medium (Sigma) supplemented with 10% inactivated fetal bovine serum (Life Technologies) and 1% (v/v) penicillin/streptomycin and maintained at 24 °C. Genomic DNA was extracted from 10^8^ promastigotes in logarithmic growth phase using a Wizard Genomic DNA Purification Kit (Promega), resuspended in DNase-free water and quantified using a NanoDrop Spectrophotometer ND-1000. The genomic DNA samples obtained from the different species of *Leishmania* were used as a template in PCR amplification reactions with selected taxon-specific primers designed by TipMT (Table 1, Additional file 1: Table S1). Each PCR used 50 ng of DNA template, 1x Green GoTaq Reaction Buffer (Promega), 200 μM dNTPs mix, 10 pmol of each forward and reverse primers, and 1.25 U of Taq DNA polymerase (Phoneutria). The samples were incubated at 94 °C for 5 min and submitted to 30 cycles of 94 °C (30 s), 63 °C (15 s) and 72 °C (10 s), followed by a final extension of 72 °C for 7 min. The PCR products were fractionated by electrophoresis in 2.5% agarose gels in TAE 1x buffer (4.8 g/L Tris-base, 1.14 mL acetic acid, 2 mL 0.5 M EDTA, pH 8.0) with 0.5 μg/mL ethidium bromide or in silver-stained 8% polyacrylamide gel in TAE 1x.

**Table 1:** List of specific (G1) and single (S1) pairs of primers for *in vitro* validation

Group	Species	Primer Name	Sequence Forward (5’ - > 3’)	Sequence Reverse (5’ - > 3’)	Amplicon (pb)
G1	*L. amazonensis*	Lam.A480-1	GCGCGCGAGAAAATAGAGAC	GGGCGTCGTCGATATCTGTT	379
	*L. braziliensis*	LbrM.11.1130-0	ACAGAACAATCAGGCCCGAG	GCTATCGGACGCCTCATCAA	244
	*L. infantum*	LinJ.36.1330-0	AGTTCCTTTGTTGTTGTGTTTCGT	TTATCTCTCCCGTCCCTCCG	334
S1	*L. infantum, L. braziliensis* and *L. major*	LmjF29-1	GCGGTGCTTGAATCACGTTT	GCGGTGTTTACATGACGACG	307-322-337

All primers were generated by TipMT on “Ortholog target” mode using *L. amazonensis, L. braziliensis* and *L. infantum* genomes (G1 primers), or *L. braziliensis, L. major* and *L. infantum* genomes (S1 primers)

## Results and discussion

### TipMT Output

#### Primers set

Selected pairs of primers are classified in one of the four categories: specific, multiple, single and conserved. After the user chooses the categories, information regarding the primer characteristics is reported, such as primer sequence, melting temperature range (°C) and PCR product length (bp). Additionally, users can choose a set of primers or save the primer information in a text file or visualize a virtual gel electrophoresis. The text file shows the following amplicon properties: 1) name; 2) forward primer sequence; 3) reverse primer sequence; and 4) primer melting temperature and amplicon size in each target genome. A list of the compatible pairs of primers that are optimal for multiplex PCR is also available to download.

#### Virtual gel

**Fig. 2:**
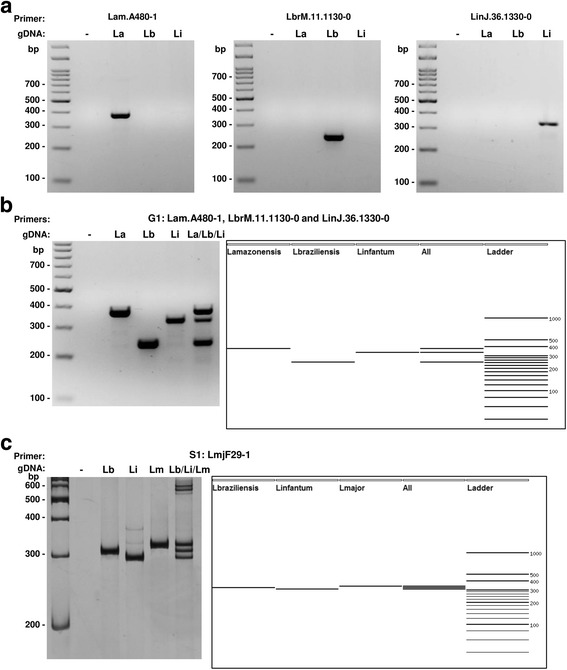
Real and virtual (e-MPX) gel electrophoresis for specific G1 pairs of primers (**a** and **b**) and single S1 pair of primers (**c**). Each lane corresponds to the combination of genomic DNA of *Leishmania* species and pair of primers identified at the top of the gel (standard PCR in **a** and **c** and multiplex PCR in **b**). La: *L. amazonensis*; Lb: *L. braziliensis*; Li: *L. infantum*; Lm: *L. major*; gDNA: genomic DNA; bp: base pair

After selecting a set of pair of primers, the user may choose to generate a virtual electrophoresis gel, a visual output with a simulated result of a conventional PCR e-GEL or multiplex PCR e-MPX (Fig. 2). In both cases, TipMT takes the set of PCR products and calculates their respective migration distances based on the length of the amplicons.

In the e-GEL function, the visual output is a simulated conventional PCR assay, where each lane is a reaction with one selected primer and a mixture of target genomes as a template. The e-MPX function generates another virtual gel with the amplification profile of the multiplex PCR assay, where each lane is a mixture of all selected primers and one target genome or all target genomes as the template.

Multiplex PCR is a variant of PCR, which simultaneously amplifies many loci of interest in one single reaction by using more than one pair of primers. Setting up a multiplex PCR with consistent quality is not trivial; therefore, TipMT generates a file with groups of specific primers that are compatible in a multiplex assay, based on primer–primer interactions and differences in the melting temperatures.

### Experimental Validation

*Leishmania* is a genus of flagellate protozoan that cause a broad spectrum of diseases, ranging from self-limiting localized cutaneous lesions to visceral leishmaniasis. More than 20 species of *Leishmania* cause infection in humans [24]. Despite the wide taxonomic complexity of this genus, the gold standard for diagnosing *Leishmania* infections, parasitological assays, only discriminates genus, not species. The reference method for species identification is multilocus enzyme electrophoresis (MLEE). However, this method has several limitations, including the relatively small number of characterized loci and the requirement of a parasite culture that potentially biases the results [25]. Therefore, the development of new molecular diagnostic methods could allow for a rapid and accurate diagnosis of *Leishmania* species in infection. This is particularly important in the context of leishmaniasis, since the distinction between *Leishmania* species is important to provide the appropriate treatment and design the most effective control measures. This specific identification is especially relevant in areas where different species occur simultaneously causing human disease. Finally, robust molecular markers might contribute to the characterization of parasite-specific features, such as virulence or drug resistance [26].

We thus applied TipMT pipeline to the genomes of different species of *Leishmania* to generated sets of primers for genotyping using Multiplex PCR. First, we tested the pipeline using *L. amazonensis*, *L. braziliensis* and *L. infantum* genomes as templates on “Ortholog target” mode and among all pair of primers generated, we chose the G1 set for *in vitro* validation (Table 1, Fig. 2). Because in this search no pair of primer was classified as single, we re-ran the pipeline using *L. braziliensis*, *L. major* and *L. infantum* on “Ortholog target” mode and we obtained the pair of primers S1 which was also used in *in vitro* experiments (Table 1, Fig. 2c). We also generated pairs of primers using *L. braziliensis* and *L. infantum* genomes on SSR (R1, R2 and R3 groups) or Ortholog (O1, O2 and O3 groups) target modes and selected 12 pairs of primer for *in vitro* validation (Additional file 1: Table S1, Additional file 2: Figure S1).

The PCR gel electrophoresis profile revealed that the result of the real experiment was the same as that predicted by the virtual electrophoresis gel analysis (Fig. 2 and Additional file 2: Figure S1).

### Comparison to other primer design applications

Our tool was developed to receive two or more genomic data and output specific pairs of primers for each taxon analyzed. So, we compared TipMT to other similar web-based primer design tools that have similar characteristics: freely available, web-based and able to find specific pairs of primers from multiple sequences. TipMT was compared to BatchPrimer3 [27], jPCR [28], MPprimer [29]. A comparison of the main features of these tools is shown in Table 2.

**Table 2:** Main features of similar web-based primer design tools

Tool	Sequence input	Search for Target sequences	Specificity check method	Output	Multiplex check
BatchPrimer3	multiple sequences (up to 500)	yes (SSR, SNP)	none	primers information	no
jPCR	multiple sequences	yes (SSR only)	alignment	primers information	yes
MPprimer	multiple sequences	no	thermodynamic (limited and pre-defined list of sequences)	primers information and virtual gel	yes
TipMT	multiple sequences from multiple taxa	yes (SSR, orthologs, singletons)	alignment and thermodynamic	primers information and virtual gel	yes

TipMT offers a combination of features that are not present in any other web application: multiple sequences as input, identifies target regions automatically in a single run, tests the specificity with two approaches and generates a virtual electrophoresis gel as output

MPprimer is a web-based tool that designs specific multiplex PCR primers, uses thermodynamic theories to estimate the stability of the primers, and has functions for predicting a group of compatible multiplex primers and generating a virtual electrophoretic gel for each group. The main limitations of MPprimer are a limited and pre-defined list of genome databases (model organisms) provided by the tool that are used for checking primer specificity and the lack of a mechanism to search for target sequences, which should then be pre-defined by the user. BatchPrimer3 allows users to design several types of primer, including generic primers, hybridization oligos, primers for SSR regions, SNP genotyping primers and DNA sequencing primers. The main drawbacks of the tool are: limited number of sequences (up to 500) that can be used for primer design; no step for checking primer specificity; and the tool does not design primers for multiplex PCR. jPCR (FastPCR online) provides primers for most PCR applications and tests the specificity using a quick local alignment screen between the reference database (user’s database sequences) and input sequence. However, the application is platform dependent (Java Runtime Environment), requires computational resources of the user and more computing power for large databases. Also, jPCR only searches for SSR as target sequences.

TipMT offers a combination of features that are not present in any other available web applications. TipMT receives multiple sequences as input and identifies target regions automatically. Moreover, primer specificity is tested by both alignment- and thermodynamic-based properties. Furthermore, TipMT provides functions to generate a virtual electrophoresisgel for conventional or multiplex PCR assays. This output gives users a visual result before performing a real PCR reaction. Finally, the identification of taxa specific pairs of primers from multiple genomic sequences is a straightforward analysis only in TipMT, since it is the only available tool that can find pairs of primers for multiple taxa in a single run using genomic data without prior definition of the target region.

## Conclusions

The emergence of large-scale DNA sequencing projects in recent years has produced large amounts of data, opening many opportunities for genomic analyses. Here, we focus our attention on identifying molecular markers and designing efficient primers for taxa differentiation. The ideal pair of primers should be capable of distinguishing the target taxon and should not cross-react with other closely related species. Toward this aim, TipMT receives genomic sequences as input and integrates the process of primer design, from the search for target sequences to the evaluation of primer specificity. As an output, the web-application generates a plain text file with general information on the pairs of primers, based on taxa-specificity. The output also includes an image showing the result of a simulated PCR assay with selected pairs of primers. Finally, experimental validation shows the effectiveness of the proposed tool in finding a taxon-specific pair of primers.

The pairs of primers generated by TipMT are suitable for use in conventional or multiplex PCR assays, as determined by the parameter settings during the primer design step. Furthermore, primer design principles for conventional PCR and real-time quantitative PCR are quite similar. Thus, our tool could be used for designing primers for both methodologies by adjusting some parameters, such as PCR product size.

Future versions of TipMT will consider multi-copy genes as targets to improve PCR sensitivity and will also receive raw sequencing reads as input. Additional improvements may also be performed, for example, automatic ranking of ideal primers for multiplex PCR.

The application is platform independent, freely available and has a simple and user-friendly interface that allows for designing primers in a high-throughput manner, even for novice users. Furthermore, TipMT web page has a “Manual” section with a tutorial and running examples. TipMT can be applied to a broad spectrum of research topics including both molecular diagnostic and evolutionary studies.

## Availability and requirements

Project name: TipMT, Tool for Identification of Primers for Multiple Taxa

Project home page: http://200.131.37.155/tipMT/

Operating system(s): Platform independent

Programming language: PHP, JavaScript, PERL

Other requirements: Web browser (supported browsers: Firefox, Chrome)

Any restrictions to use by non-academics: no license needed

## Additional files

Additional file 1: Table S1. List of specific pair of primers for *in vitro* validation. All primers were generated by TipMT using *L. braziliensis* and *L. infantum* genomes on “SSR target” mode (R1, R2 and R3) or “Ortholog target” mode (O1, O2 and O3). (DOCX 14 kb)
Additional file 2: Figure S1. Real and virtual (e-MPX) gel electrophoresis for SSR (A) and Orthologs (B) primers. Each lane corresponds to the combination of genomic DNA of *Leishmania* species identified at the top and a mixture of the orthologs (O1, O2 and O3) or SSR (R1, R2 and R3) primers in a multiplex PCR assay. Lb: *L. braziliensis*; Li: *L. infantum*; gDNA: genomic DNA; bp: base pair. (TIFF 1741 kb)

## Abbreviations

BLAST: Basic local alignment search tool
HTML: Hypertext markup language
PHP: PHP hypertext preprocessor
RefSeq: Reference sequence
