# Supplementary material for: TipMT: Identification of PCR-based taxon-specific markers
Source: BMC Bioinformatics. 2017 Feb 11;18:104. doi: 10.1186/s12859-017-1485-3 (PMC5303226; doi:10.1186/s12859-017-1485-3)
Supplement: Additional file 1: Table S1. — List of specific pair of primers for in vitro validation. All primers were generated by TipMT using L. braziliensis and L. infantum genomes on “SSR target” mode (R1, R2 and R3) or “Ortholog target” mode (O1, O2 and O3). (DOCX 14 kb) [file 12859_2017_1485_MOESM1_ESM.docx]

Title: List of specific pair of primers for *in vitro* validation

| **Group** | **Species** | **Primer Name** | **Sequence Forward (5’ -> 3’)** | **Sequence Reverse (5’ -> 3’)** | **Amplicon (pb)** |
| --- | --- | --- | --- | --- | --- |
| R1 | *L. braziliensis* | LbrM.20.1-1 | CGTGAAGCTGCTTGGCAAAA | AGTGGTGGTGTGCGAAAAGA | 225 |
|  | *L. infantum* | LinJ.14-2 | GCTTCGAGGCTAACCCGATT | CACTCGCCTTTCCGCTATCT | 289 |
| R2 | *L. braziliensis* | LbrM.03-1 | AGCCATCGCTCACTAGAAGC | CCTTCCGTGATGCCAGGTAA | 275 |
|  | *L. infantum* | LinJ.08-1 | TCGATAACTGCACAGCTCGT | TGTGTGTGCTTGTGGCTCAT | 301 |
| R3 | *L. braziliensis* | LbrM15 | TGTTTGGCTTTCTGGCTACA | CACCCACACAGTGACACACA | 254 |
|  | *L. infantum* | LinJ09-12 | AAGATGAAGCTCCTCCGTCA | CCGACTTCGTCCGTTATTCA | 302 |
| O1 | *L. braziliensis* | LbrM31-2 | CTCAGCGTCTCCTCATTGCA | TAGTTTCGCGCACCTCTGAG | 229 |
|  | *L. infantum* | LinJ32-1 | GCTTTTGCATGTCACCACGT | CGTCCATGCTACCCCTCAAG | 305 |
| O2 | *L. braziliensis* | LbrM30-1 | ATCTCGGTGGAGGGAGACAA | AGATGCCAATGGTGGGTTGT | 256 |
|  | *L. infantum* | LinJ20-0 | GAAGACGGTGGTGAGAGTGG | CTCTTCAAGGGTGCCCAGAG | 323 |
| O3 | *L. braziliensis* | LbrM34 | CACCCCAAAAGAATCCAGAA | CTCTTTAGTGGATCAGCGCC | 250 |
|  | *L. infantum* | LinJ26 | CTTTGATAACATCACCGCCC | CCAAGTTTCTGCAGGTCCTC | 306 |

**Table S1**: List of specific pair of primers for *in vitro* validation. All primers were generated by TipMT using *L. braziliensis* and *L. infantum* genomes on “SSR target” mode (R1, R2 and R3) or "Ortholog target" mode (O1, O2 and O3).
