# Supplementary figures and images for: TipMT: Identification of PCR-based taxon-specific markers
Source: BMC Bioinformatics. 2017 Feb 11;18:104. doi: 10.1186/s12859-017-1485-3 (PMC5303226; doi:10.1186/s12859-017-1485-3)

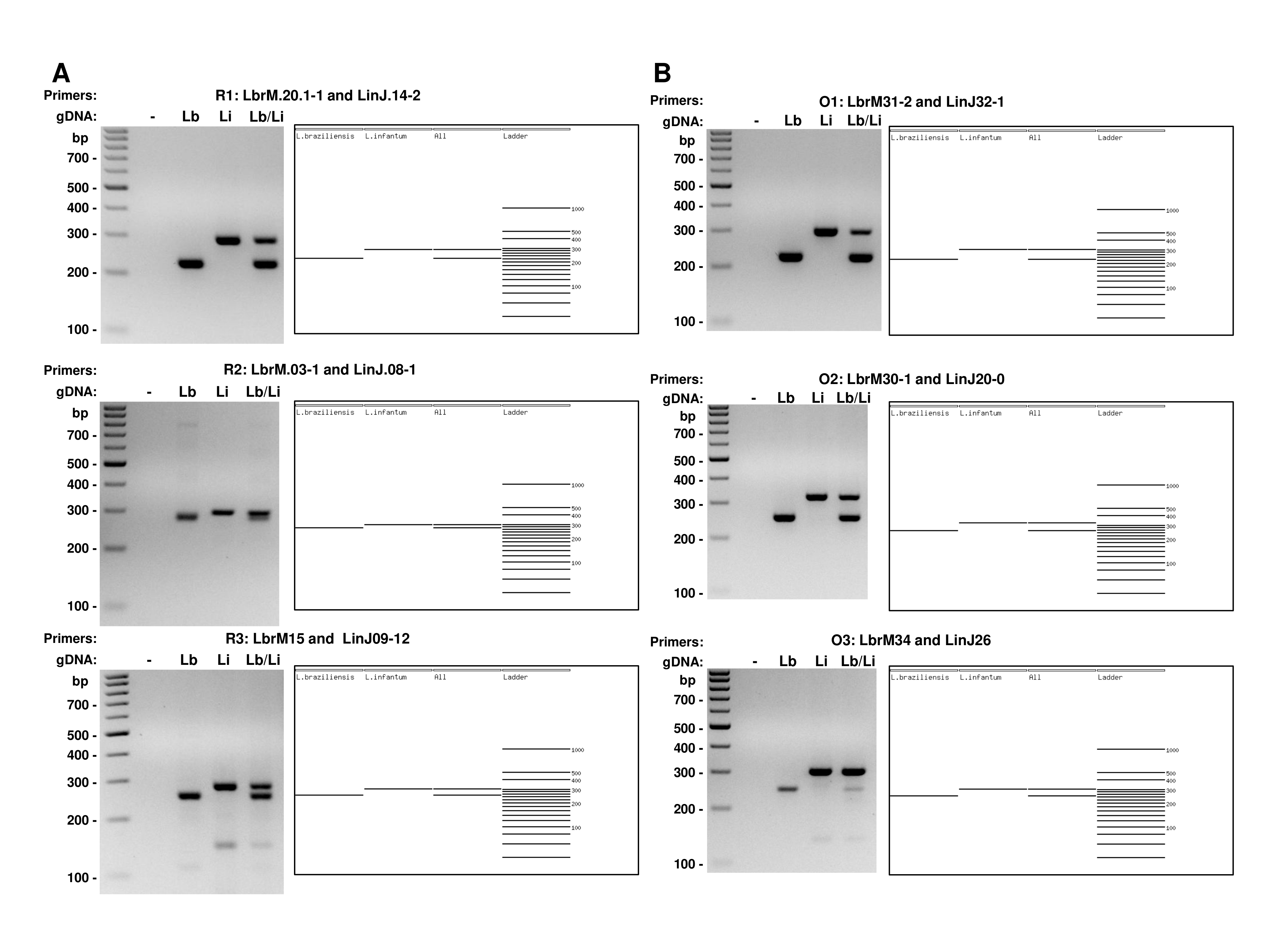

Supplement: Additional file 2: Figure S1. — Real and virtual (e-MPX) gel electrophoresis for SSR (A) and Orthologs (B) primers. Each lane corresponds to the combination of genomic DNA of Leishmania species identified at the top and a mixture of the orthologs (O1, O2 and O3) or SSR (R1, R2 and R3) primers in a multiplex PCR assay. Lb: L. braziliensis; Li: L. infantum; gDNA: genomic DNA; bp: base pair. (TIFF 1741 kb) [file 12859_2017_1485_MOESM2_ESM.tiff]
